# *Helicobacter pylori* Antibody Titer and Gastric Cancer Screening

**DOI:** 10.1155/2015/156719

**Published:** 2015-09-30

**Authors:** Hiroshi Kishikawa, Kayoko Kimura, Sakiko Takarabe, Shogo Kaida, Jiro Nishida

**Affiliations:** Department of Gastroenterology, Tokyo Dental College, Ichikawa General Hospital, 5-11-13 Sugano, Ichikawa, Chiba 272-8513, Japan

## Abstract

The “ABC method” is a serum gastric cancer screening method, and the subjects were divided based on *H. pylori* serology and atrophic gastritis as detected by serum pepsinogen (PG): Group A [*H. pylori* (−) PG (−)], Group B [*H. pylori* (+) PG (−)], Group C [*H. pylori* (+) PG (+)], and Group D [*H. pylori* (−) PG (+)]. The risk of gastric cancer is highest in Group D, followed by Groups C, B, and A. Groups B, C, and D are advised to undergo endoscopy, and the recommended surveillance is every three years, every two years, and annually, respectively. In this report, the reported results with respect to further risk stratification by anti-*H. pylori* antibody titer in each subgroup are reviewed: (1) high-negative antibody titer subjects in Group A, representing posteradicated individuals with high risk for intestinal-type cancer; (2) high-positive antibody titer subjects in Group B, representing active inflammation with high risk for diffuse-type cancer; and (3) low-positive antibody titer subjects in Group C, representing advanced atrophy with increased risk for intestinal-type cancer. In these subjects, careful follow-up with intervals of surveillance of every three years in (1), every two years in (2), and annually in (3) should be considered.

## 1. Introduction

Clinicians usually regard the results of* H. pylori* serology as a categorical variable (i.e., positive or negative), while not considering the actual titer of anti-*H. pylori *antibodies. Although the antibody titer itself suggests little clinically useful information in individual cases, subjects at high risk for gastric cancer can be detected effectively by evaluating antibody titer results in populations stratified by the degree of gastric mucosal atrophy. Many investigators have used the “ABC method,” combining* H. pylori* antibody titers and serum pepsinogen (PG) concentrations, to evaluate the individual grade of atrophy and cancer risk [1–4]. Typically, the significance of a serum screen for the measurement of* H. pylori* antibody titer has been discussed in the context of the ABC method. For example, “high-positive antibody titer” subjects exhibit increased risk of diffuse-type gastric cancer compared to populations without gastric atrophy, and “low-positive antibody titer” subjects exhibit increased risk of differentiated adenocarcinoma in populations with gastric mucosal atrophy. These seemingly contradictory results have been confirmed by several investigators, and the scientific basis of these results has also been analyzed in detail [5–8]. However, these data have been known only to a limited number of investigators and clinicians and have not been widely disseminated.

In the present review, we first describe the characteristics of* H. pylori* antibody titers in the context of screening for* H. pylori* infection, including consideration of the biological meaning of the serum anti-*H. pylori* antibody titer. We then discuss several reported results concerning anti-*H. pylori* antibody titers. These data suggest the use (in daily clinical practice) of an expanded ABC method to detect patients with elevated risk for gastric cancer.

## 2. Characteristics of the* H. pylori* Antibody Titer as a Screening Method for* H. pylori *Infection

Measurement of the serum anti-*H. pylori* antibody titer is a noninvasive, inexpensive, and readily available method for detection of* H. pylori* infection. Histology, culture, polymerase chain reaction (PCR), and the rapid urease test all require biopsy and/or collection of specimens by endoscopy, an invasive technique that is not suitable for mass screening [9, 10]. The urea breath test and stool antigen test are regarded as noninvasive tests, but the results of both methods are significantly affected by proton pump inhibitor therapy [11–13]. However, validated serology tests can be used even in patients being treated with proton pump inhibitors.

*H. pylori* strains possessing the cytotoxin-associated gene A (CagA) protein, a well-known virulence factor, cause more extensive inflammation and severe atrophy in gastric mucosa than nonproducers [14, 15]. However, there is still controversy regarding the significance of CagA serology, especially in East Asia, where most strains of* H. pylori* are CagA producers [16–19]. Therefore, gastric cancer screening is usually performed using the* H. pylori* antibody titer alone, except in limited areas [20].

Burucoa et al. [21] investigated the accuracy of 29 different serological tests and reported positive and negative predictive values of 70% and 100%, respectively. In general, better performance in serological screening depends on the use of the appropriate antigens and adjustment of cut-off values [22]. These considerations are among the disadvantages of using serum* H. pylori* antibody as a screening test for gastric cancer. Another disadvantage of using* H. pylori* antibody is that serology alone presents a challenge in distinguishing past and current infections [23]. The use of serology to identify posteradicated cases is considered later in this review.

## 3. The ABC Method: Gastric Cancer Screening Using* H. pylori* Antibody Titer and Pepsinogen Levels

High seropositive rates for* H. pylori* antibody are observed in East Asia, Eastern Europe, and parts of Central and South America, all areas with populations that have high levels of gastric cancer. Thus, in these regions, the anti-*H. pylori *antibody titer alone is insufficient for use as a screening tool for gastric cancer risk [24, 25]. For example, the frequency of anti-*H. pylori* seropositivity in subjects born before 1950 is reported to be over 70% in north Japan [26].

PG protein is classified into two types (PGI and PGII) based on biochemistry and immunology. PGI is produced by the chief cells in the gastric body; PGI levels decrease proportionally with the progression of atrophy of the gastric body. PGII is secreted by most parts of the gastric mucosa, as well as by parts of the duodenum; PGII levels increase in association with gastric mucosal inflammation regardless of the degree of atrophy [27–29]. As a result, atrophic gastritis can be diagnosed serologically by assaying the serum levels of the two pepsinogen isozymes. Reductions in serum PGI levels and in the serum PGI/II ratio are reliable markers for atrophic gastritis; cut-off values of PGI ≤70 ng/mL and PGI/II ratio ≤3 have frequently been applied [30–32].

The European Society of Gastrointestinal Endoscopy has proposed PG levels as a predictor of extensive atrophic gastritis [33]. Miki reported that the odds ratio (OR) for death from gastric cancer within 3 years after screening by the PG method was 0.290 [1]. Several other well designed, large-cohort studies have suggested an association between PG levels and dysplasia/gastric cancer. Watabe et al. found that* H. pylori*-positive patients with PGI <70 and PGI/II ratio <3 had a hazard ratio (HR) for gastric cancer of 6.0 [2]; under the same conditions, Yamaji et al. reported an HR of 6.2, and Yanaoka et al. reported an HR of 2.77 [34, 35]. The preceding studies corresponded to Japanese populations, but a report from Portugal showed consistent results in Western individuals [36]. However, gastric cancers did develop in a fraction (approximately 40%) of individuals who were atrophy-negative as defined by PG levels; the pathological characteristics of such cases revealed an unexpectedly high percentage of diffuse cancer [35].

About half of the cases of diffuse cancer, of which the prognosis is sometimes poor, cannot be detected by PG methods. This observation represents a critical weak point in mass screening by the PG method alone. Thus, a combined analysis (the “ABC method”) incorporating the anti-*H. pylori* antibody assay and mucosal atrophy (as determined by serum PG) has been proposed to stratify the risk for the development of gastric cancer [1–4, 37]. Notably, the ABC method permits detection of gastric cancer in PG-negative subjects, addressing the fact that most diffuse cancer is* H. pylori*-positive but PG-negative. According to the original ABC method (as proposed by Miki [1]), PG was defined as “atrophic” when the criteria of both PGI ≤ 70 ng/mL and PGI/II ≤3 were fulfilled. Under the ABC method, the subjects were divided into the following 4 groups according to the presence of atrophic gastritis identified by serum PG and* H. pylori* seropositivity: Group A [*H. pylori *(−) PG (−)], Group B [*H. pylori* (+) PG (−)], Group C [*H. pylori* (+) PG (+)], and Group D [*H. pylori* (−) PG (+)]. Subjects in Groups B, C, and D were advised to undergo* H. pylori* eradication and endoscopic screening. The recommended endoscopy intervals in Groups B, C, and D are every three years, every two years, and every year, respectively, because the risk of gastric cancer was highest in Group D, followed by the risks in Groups C, B, and A [1, 4, 35]. Mizuno et al. reported (using Group A as a reference) that the HR of gastric cancer in Group B was 4.2, and those of Groups C and D were 11.23 and 14.81, respectively [4]. The ABC method is a screening strategy of “risk stratification” to identify high-risk subjects. Using the ABC method, we can advise high-risk subjects to undergo (1) regular endoscopic surveillance and (2) eradication of* H. pylori* infection. Subjects with a past history of* H. pylori* eradication were strictly excluded from screening by the ABC method; specifically, a subset of cases with successful eradication showed negative antibody titers in combination with increased PGI/II ratios, thus rendering “false-negative” results [HP (−) PG (−)] in most eradication cases. Kudo et al. reported that only 10.6% of all gastric cancer patients would have been classified into Group A, indicating that approximately 90% of cancer subjects would have been regarded as positive by the ABC method [3].

Despite the apparent relationship between serum PG and extensive atrophic gastritis, the association between endoscopic atrophic grade and histological diagnosis of atrophic gastritis remains controversial. Several investigators have concluded that conventional white-light endoscopy cannot accurately differentiate and diagnose atrophic gastritis [38, 39]. This inconsistency suggests that endoscopic findings are insufficient for the accurate prediction of the grade of atrophy.

The essential weakness of the ABC method is the fact that remarkably high percentages of subjects are classified into Groups B–D, for whom endoscopy is indicated. One study suggested that only 45–60% of Japanese subjects in their 50s and 60s would be classified as Group A [26]. This limitation should be recognized when using this method for gastric cancer screening, especially in populations with high endemic rates of* H. pylori* infection.

The other limitation of the ABC method is that heterogeneity of risk in each subgroup has been reported by several investigators. All subjects in Group A do not show the same risk for gastric cancer; the high-risk subjects in Group A can be detected by stratification using antibody titers, and the same heterogeneity is seen in Groups B and C. We will refer to the details of the association between antibody titer and risk stratification of each subgroup in the ABC method in the following section.

## 4. Significance of the* H. pylori* Antibody Titer in the ABC Method

### 4.1. Associations between Anti-*H. pylori* Antibody Titer and the Degree of* H. pylori* Colonization

The immunopathological response induced by* H. pylori* infection is hypothesized to be dose-dependent. Higher bacterial counts induce intense immune response, resulting in subsequent higher antibody titers. However, genetic differences of each human host may affect antibody levels to pathogens [40]. Immune suppression by advanced cancer is another conceivable factor affecting the antibody titer of* H. pylori*, although direct proof of immune suppression in relation to the progression of gastric cancer has not yet been demonstrated [41]. Nonetheless, several investigators have inferred significant associations between the anti-*H. pylori* antibody titer and* H. pylori* density. Several investigators have found that the serological absorbance index of IgG antibodies against* H. pylori *is related to the density of antral* H. pylori* colonization and polymorphonuclear cell infiltration [42, 43]. The observation of significant decreases in* H. pylori* antibody titers following successful eradication implies the existence of a quantitative relationship [44]. Marchildon et al. reported that the mean decrease in IgG titer of* H. pylori* antibody at 6 months is approximately 40% [45]. Considered together, these findings indicate that the* H. pylori *antibody titer might be used to estimate the density of* H. pylori* in whole gastric mucosa.

### 4.2. Significance of “Low” Antibody Titer of* H. pylori* in Gastric Cancer Screening

#### 4.2.1. Spontaneous Disappearance of* H. pylori* after Progression of Atrophic Gastritis in ABC Method-Defined Groups C and D

Chronic infection by* H. pylori* leads to* H. pylori*-related gastritis, which starts in the antrum and expands proximally towards the gastric body [46, 47]. Several investigators analyzing the association between gastric cancer development and* H. pylori* antibody titer demonstrated that this association is not proportional [35, 48, 49]. Multiple studies have reported that gastric cancer risk is elevated in subjects who display low-titer seropositivity for* H. pylori* antibody in combination with advanced mucosal atrophy (as determined by the PG level) (i.e., Group C in the ABC method) compared to high-titer subjects [5, 35, 50, 51]. Yanaoka et al. first referred to the significance of a “low-positive titer” in screening for gastric cancer. Those authors reported that the hazard ratio (HR) of overall cancer incidence in low-positive antibody titer subjects with serological atrophy was significantly higher (HR = 11.4) than in those with high-positive antibody titers (HR = 6.7), suggesting an increased risk in “low titer” patients, especially atrophic subjects [35]. Based on a case-controlled study, Fujioka et al. reported that the median serum antibody titer was lower in cancer cases than in control cases, such that high-positive titer patients have a reduced risk for gastric cancer (OR = 0.39) [50]. Note, however, that that study population was not confined to subjects with atrophy (i.e., not limited to Group C only). In a large study (with >36,000 participants), Tatemichi et al. clearly demonstrated an association between a low-seropositive* H. pylori* antibody titer and a high incidence of gastric cancer among PG-positive subjects [5]. That paper reported that, among the severely atrophic subjects in Group C, the ORs of cancer incidence (using Group A as reference) in low-positive titer and high-positive titer subjects were 14.9 and 8.3, respectively, suggesting that the risk in low-positive titer cases was almost double that in high-positive titer cases. That paper also analyzed the risk of intestinal- and diffuse-type cancers in “low-positive antibody titer” patients, demonstrating a significant association between the risk of cancer and “low-positive antibody titer” in intestinal-type cancer, but not in diffuse-type cancer.

To make sense of these results, we need to understand the environment suitable for survival of* H. pylori *in gastric mucosa.* H. pylori* can survive in gastric epithelial cells; thus, the loss of gastric epithelial cells after the progression to advanced atrophic gastritis with intestinal metaplasia can lead to spontaneous elimination of* H. pylori* [52, 53]. Thus, after infection with* H. pylori *in childhood, the bacterial density is expected to increase; presumably during this period, atrophy is limited to the antrum. Subsequently, a subset of these cases will progress to further atrophy of the gastric corpus; in these individuals,* H. pylori* numbers will gradually decrease, with some of these cases developing intestinal-type cancer in accordance with Correa's hypothesis [46]. In our experience, approximately 20% of subjects older than 60 years who are classified as Group C (by the ABC method) show low-positive* H. pylori* antibody titers (data not shown). This observation suggests that most subjects in Group C do not show progressive atrophy. Thus, for Group C patients, an elevated risk of gastric cancer development is indicated by the combination of both low* H. pylori *antibody titer and mucosal atrophy.

We have also focused on the role of antibody titer, with data suggesting the significance of “low-positive titer” in subjects with atrophy. Specifically, our investigation of the association between the* H. pylori* antibody titer and fasting gastric pH revealed that impairment of acid secretion (a functional indicator of gastric atrophy) was most severe in patients with atrophic mucosa [PG(+)] and low-positive* H. pylori* antibody titer (i.e., Group C as defined by the ABC method) [54]. Our findings suggest that these subjects have an extremely high risk for gastric cancer, an interpretation that is consistent with previous reports, including Tatemichi et al. [5]. These results may also explain the occurrence of gastric cancer in ABC-defined Group D [PG(+), HP (−)]:* H. pylori* may be “disappearing” from the mucosa in a state of advanced atrophy [5]. Presumably, when the antibody titer in Group C fell below the cut-off value, those cases were classified as Group D (seronegative). Comparison of severely atrophic* H. pylori*-negative cases (Group D) to less atrophic* H. pylori*-positive cases (Group C) yielded respective HR values in gastric cancer of 131.98 versus 2.77 in Yanaoka et al. [35] and of 61.85 versus 14.85 in Ohata et al. [55]. The European Society of Gastrointestinal Endoscopy also noted that the spontaneous disappearance of* H. pylori* antibody was associated with the progression to severe gastric mucosal atrophy [33].

Consequently, the consensus of opinion now holds that subjects harboring the combination of low-positive* H. pylori* antibody titer and atrophic gastric mucosa are at higher risk for gastric cancer than subjects with a high-positive antibody titer, especially intestinal-type cancer. We recommend the following two management steps for these subjects: eradication of* H. pylori* and short-interval endoscopic surveillance. Eradication of* H. pylori* is usually advised in the ABC method; however, we recommend reconfirming it, especially in subjects with a low-positive antibody titer in Group C. In Group D, infection of* H. pylori *should be strictly investigated using other methods (urea breath test, stool antigen, etc.), and it should be eradicated if positive. Based on the ABC method, the interval for endoscopy is every two years in Group C and every year in Group D; however, we consider it desirable for patients with a low-positive antibody titer in Group C to undergo endoscopy every year, based on the result of Tatemichi et al. that the HR of gastric cancer in subjects with a low-positive antibody titer is twice that of those with a high-positive antibody titer.

Because the normal value of* H. pylori* antibody titer is different with each EIA kit, optimization of the cutoff value used to divide the “low-positive” and “high positive” subjects should be considered. Previous reports by Tatemichi et al. [5] and our own [54] set the cutoff point to 57.7 U/mL and 50 U/mL for the low-positive and high-positive antibody titer groups using the EIA kit of E plate, Eiken, ensuring the ratio of both group becomes about half of all* H. pylori-*seropositive subjects. Thus, we advocate that* H. pylori-*seropositive cases should be subdivided into two groups, low-positive and high-positive groups, and the cutoff value should be set to the points at which about one half of* H. pylori-*seropositive subjects are classified into each group.

#### 4.2.2. Disappearance of* H. pylori* after Unexpected Eradication by Antibiotics and Its Significance in Group A

Dissemination of antimicrobial resistance induced by inappropriate use or abuse of antibiotics is a global public health problem [56]. The reported rate of inappropriate antibiotic use in hospitals ranges from 26% to 57% [57, 58], and the adherence rates for drug selection and treatment duration are relatively low, with rates as low as 38% even in Japan [59]. The failure of conventional triple therapies to eradicate* H. pylori* has been attributed primarily to bacterial resistance to one of the most commonly used antibiotics, clarithromycin [60–62]. The prevalence of clarithromycin resistance in the USA has increased from 9.1% in 2009-2010 to 24.2% in 2011–2013 [57]. In Italy, the prevalence of clarithromycin resistance has increased rapidly, doubling from 10.2% to 21.3% between 1990 and 2005 [62]. The rate of clarithromycin resistance at younger ages (in individuals under 30 years) was reported to be as high as 57% in Japan and 7.4% in USA [63, 64].

These findings suggest the possibility that a large fraction of* H. pylori* subjects achieved unexpected and complete* H. pylori *eradication after exposure to antibiotics for the treatment of other infectious diseases. The ABC method specifies the exclusion of subjects with a past history (as determined by medical interview) of eradication of* H. pylori* infection. However, subjects with unexpected eradication cannot be excluded completely, and such subjects are at risk for developing cancer as a result of a previous* H. pylori* infection.

The Shandong Intervention Trial is the first single trial indicating that the incidence of gastric cancer was significantly reduced in* H. pylori*-eradicated cases, with an OR of 0.6 after a follow-up period of 14.3 years [65]. Additional reports, including meta-analyses, have demonstrated that the eradication of* H. pylori *decreases the possibility of future cancer development by 30% [66–68]. These findings, however, suggest that the future cancer risk is not completely eliminated. Although several investigators have reported a low incidence of gastric cancer in subjects classified into Group A [55, 69], other investigators recently reported that approximately 2–10% of gastric cancer patients were serologically classified into “Group A.” Most of these subjects were hypothesized to be “unexpectedly and successfully eradicated cases,” given that subjects with a past history of eradication are excluded from screening by the ABC method but nonetheless have a risk of later development of gastric cancer [70–73].

The pathological characteristics of cancer in patients classified in Group A are controversial. Boda et al. reported that 92.6% of gastric cancer cases classified in Group A are intestinal-type; however, those authors investigated a limited number of early-stage subjects treated by endoscopy [70]. We also found that 90% of cancer in the subjects in Group A was the intestinal-type. Meanwhile, Kato et al. reported that only 33% of cancer cases in Group A were intestinal-type [72]. It is speculated that diffuse-type cancer arises from truly negative cases, and intestinal-type cancer arises from posteradicated cases. However, the details of the pathological characteristics of cancer in Group A will need to be investigated further. In this context, “successfully eradicated cases” are misclassified as Group A in the ABC method, and so these patients are not regarded as candidates for endoscopy, despite an elevated risk for subsequent transformation. Histological damage from undiagnosed but resolved* H. pylori* infections is hypothesized to be the main source of gastric neoplasia among members of Group A.

We have recently been investigating predictive factors associated with gastric cancer in subjects who are negative for serum* H. pylori* antibody with normal PG status (i.e., Group A). We found that high-negative antibody titer (≥3 U/mL and <10 U/mL) is the significant factor for the prediction of gastric cancer in Group A (unpublished data). Although significant decreases in antibody titer after successful eradication have been reported [44, 45], complete seronegativity (antibody titer of zero) is typically not observed. We speculate that this persistence of a nonzero titer after eradication is the reason why lowering the cut-off value of the antibody titer is effective for the detection of neoplasia in Group A. Clinicians should be aware that a subset of serologically* H. pylori*-negative subjects are not true-negative cases but eradicated cases, even in patients who deny a past history of eradication treatment. It should also be noted that there is a possibility that* H. pylori* is not completely eradicated in serologically* H. pylori*-negative subjects; eradication is necessary in such cases.

Patients with unexpected eradication can also be detected by a characteristic change in serum PG. These individuals correspond to cases previously reported to possess “increased PGI/II ratios and decreased PGI levels,” who were regarded as “normal” by the PG test [74, 75].

Based on the ABC method, subjects in Group A are not usually advised to undergo second-level diagnostic assessment using endoscopy; however, we consider that Group A subjects with a high-negative antibody titer should undergo endoscopy at least every three years, as in Group B. We also recommend investigating the* H. pylori* infection status in these subjects, and if* H. pylori* is detected by methods other than serology (urea breath test or stool antigen), we consider that it should be eradicated.

We advocate that* H. pylori* seronegative cases should be divided into two subgroups, low-negative and high-negative groups, with the ABC method. Optimization of the cut-off value should be considered to identify subjects with a “high-negative”* H. pylori* antibody titer, which corresponds to 3 U/mL (3 U/mL is the minimum determination limit and 10 U/mL is the recommended cutoff point) when using E plate, Eiken in our examination. We recommend resetting the cut-off value to the lower limit of each EIA kit and to evaluate the ratio of neoplasia cases in group A defined by strict criteria.

#### 4.2.3. Classification of Subjects with Negative* H. pylori* Antibody Titers May Be Assisted by Serological Characterization of the Titer

Given the influence on the* H. pylori* antibody titer of the above-mentioned mechanisms, we propose classifying subjects negative for* H. pylori* antibody titer into the following three categories: (1) truly infection-negative; (2) infection-positive with deep progression of atrophic gastritis (in which antibody titer was normalized following spontaneous disappearance of* H. pylori* from the gastric mucosa); and (3) infection-positive, in which infection has been successfully eradicated. The eradicated cases (Class 3) are further subdivided into two categories: subjects in whom infection is eradicated by conventional triple therapy and subjects in whom infection is unexpectedly eradicated without a past history of eradication therapy.

Class 1 (infection-negative) individuals are “true-negative” cases. Most infection-negative cases will exhibit normal PG levels, and so would be categorized as Group A [*H. pylori* (−) PG (−)] according to the ABC method. Members of Class 1 typically will exhibit high PGI levels and are at extremely low risk for future gastric carcinogenesis.

Class 2 (infection-positive with deep progression of atrophic gastritis) cases will exhibit normalized antibody titers and PG-positive status, and so would be categorized as Group D [*H. pylori* (−) PG(+)] according to the ABC method. Members of Class 2 are at extremely high risk for intestinal-type gastric carcinogenesis.

Class 3 (infection-positive but successfully eradicated) cases will yield normal PG levels, and so would be categorized as Group A [*H. pylori* (−) PG (−)] according to the ABC method. Members of Class 3 have a chance of developing intestinal-type gastric cancer.

### 4.3. Significance of “High” Antibody Titer of* H. pylori* in Gastric Cancer Screening

About 20–30% of stomach cancers in Western countries develop from nonatrophic stomach, and the major histopathological type of these cancer cases has been reported to be “diffuse-type” [76, 77]. The most significant characteristic of diffuse-type cancer is a higher malignant potential than the intestinal type. The inflammation of gastric mucosa induced by* H. pylori* infection is postulated to directly induce this diffuse-type cancer without passing through the well-known sequence of atrophy to metaplasia to dysplasia to cancer. Inflammation-induced DNA methylation has been implicated in the induction of diffuse-type gastric cancer [78]. Consequently, diffuse-type cancer typically does not show advanced atrophy, and this is the reason why PG level, a reliable marker of gastric atrophy, cannot detect this type of cancer effectively. Thus, the risk factors for developing cancer among nonatrophic stomach cases include high PGII level and high* H. pylori* antibody titer, both of which reflect active gastric mucosal inflammation [79].

The association between diffuse-type cancer and a “high-positive” titer* H. pylori *antibody has been reported by several investigators. Tatemichi et al. conducted a case-controlled study using gastric cancer cases and matched control subjects, and they demonstrated that high-titer patients have the highest risk for diffuse cancer development [80]. Watanabe et al. recently elucidated, for the first time, the risk for diffuse-type gastric cancer in PG-negative individuals with high-titer* H. pylori* antibody (ABC method Group B). By performing annual endoscopies in* H. pylori*-positive subjects who did not exhibit chronic atrophic gastritis (i.e., Group-B subjects), Watanabe et al. demonstrated that cancer incidence was significantly higher in the* H. pylori* high-titer group than in the low-titer group, obtaining an HR of 6.51 [7]. That study also reported a marked increase in cancer development (at 1524/100,000 person-years) in Group B subjects harboring high-titer antibody and high PGII levels (PGI >50 ng/mL and PGI/II ratio ≤3). Other investigators have reported similar results [35]. Yoshida et al. recently reported that the HR values of diffuse-type cancer in high-titer subjects stratified by PGII (<30 ng/mL and >30 ng/mL) were 3.8 and 8.5, respectively, when cancer incidence in low-titer subjects with PGII <30 ng/mL was used as in [8]. In our own work, we have observed that approximately 15% of Group B subjects over the age of 60 years show high* H. pylori* antibody titers (data not shown), suggesting that most subjects in Group B do not show progressive mucosal inflammation.

The high-positive anti-*H. pylori* antibody titer in Group B represents a risk factor for future gastric carcinogenesis, especially for diffuse-type cancer. Considering the high prevalence of diffuse-type cancer in Western countries, the combination of increased* H. pylori* antibody titer and high PGII levels is expected to be of use as a serum (noninvasive) screen for gastric cancer among Western populations. Because* H. pylori* eradication reduces the cancer risk more effectively in subjects without extensive chronic atrophic gastritis [81], eradication of* H. pylori* infection in these subjects is strongly recommended for prevention of gastric cancer in these patients in Group B. Although eradication is usually advised after conducting the ABC method, we recommend reconfirming eradication in these subjects. Based on the ABC method, endoscopy every three years is recommended for the subjects in Group B; however, the HR of subjects with a high-positive antibody titer in Group B is two to three times higher than of those with high-positive titers [8], and thus the HR of cancer in these high-positive subjects is estimated to be similar to that of Group C. Thus, meticulous endoscopic surveillance should be performed every two years in Group B subjects with a high-positive antibody titer.

The cutoff value should be set so that one half of* H. pylori*-seropositive subjects are classified to each of the “low-positive” and “high-positive” groups, as referred to in Section 4.2.1. This value is 50 U/mL when using E plate, Eiken based on our investigations (10 U/mL is the recommended cutoff point).

## 5. Natural History of* H. pylori*-Positive Subjects and the Relationship with the* H. pylori* Antibody Titer

The natural history of subjects with* H. pylori* infection has usually been discussed in the context of Correa's hypothesis [46], which postulates that gastric carcinogenesis occurs as a continuous process beginning with superficial gastritis and proceeding through metaplasia and dysplasia before reaching adenocarcinoma. However, few investigators have reviewed the natural history of* H. pylori*-infected subjects from the standpoint of the* H. pylori* antibody titer profile.

Here, we present the “typical” natural history of* H. pylori*-positive subjects and the associations with* H. pylori* antibody titer. We believe that readers will gain an overview of the significance of the antibody titer in each pathophysiology by reading this section.

**Figure 1:**
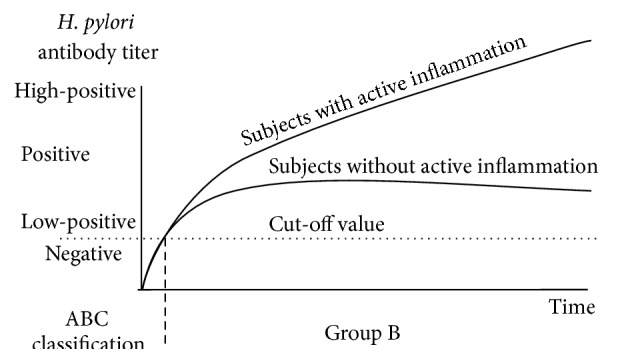
Time course of changes in the* H. pylori* antibody titer in* H. pylori*-infected subjects without significant progression of mucosal atrophy (Grade B in the ABC method). The antibody titer does not show significant change in most Group B cases. An increase in the* H. pylori *antibody titer is observed in a subset of these patients with progressive mucosal inflammation; such cases have an increased risk for diffuse-type cancer. Thus, subjects with a high-positive antibody titer should be considered at high risk for future development of diffuse-type gastric cancer.

**Figure 2:**
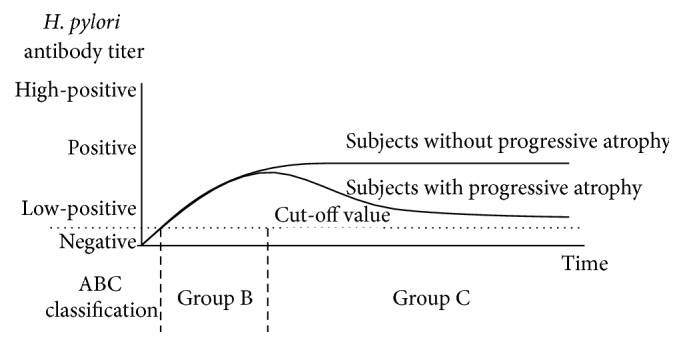
Time course of changes of the* H. pylori* antibody titer in* H. pylori*-infected subjects with progression of mucosal atrophy (Grade C in the ABC method). In Group C, the antibody titer does not change significantly (in contrast to the pattern in most Group B cases). However, in a subset of Group C cases, the antibody titer of* H. pylori* decreases gradually due to shrinkage of the effective area of the gastric mucosa as a result of the progressive atrophy induced by* H. pylori* infection. Thus, Group C subjects with low-positive* H. pylori* IgG titers exhibit an elevated risk for intestinal-type gastric cancer.

**Figure 3:**
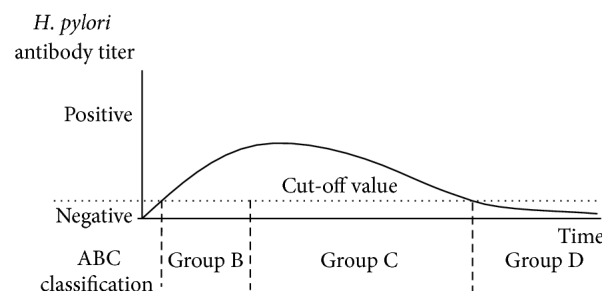
Time course of changes of the* H. pylori* antibody titer in* H. pylori*-infected subjects with severe progression of mucosal atrophy (Grade D in the ABC method). Subjects in Group D show significant “advanced” progression of atrophy; members of this group become seronegative for* H. pylori* antibody titer following the spontaneous disappearance of* H. pylori* from gastric mucosa. This subset of patients would be classified originally as Group B and then as Group C as atrophy progresses and finally as Group D when the antibody titer falls below the cut-off value (converting to antibody-negative status, as indicated by the arrow). Approximately 1% of all subjects in Japan will be classified as Group D, suggesting that only a limited number of cases show “advanced” progression of atrophy.

**Figure 4:**
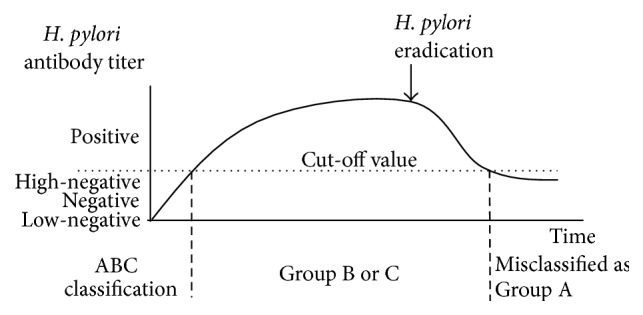
Time course of changes of the* H. pylori* antibody titer in successfully eradicated cases (classified as Grade A, although these cases should be excluded by medical interview in the ABC method). After successful eradication of* H. pylori*, the antibody titer decreases due to disappearance of* H. pylori* from the gastric mucosa, and from that point, the degree of atrophic grade progresses no further. Thus, a defined risk for gastric cancer persists throughout the lifetime of these patients, because a certain degree of gastric mucosal atrophy exists even after infection eradication. Therefore, eradicated cases should not be included as candidates in the ABC method, and they will require surveillance by endoscopy.

Figures 1–4 show a schematic view of the four patterns of the serial change of* H. pylori* antibody titer.

Figure 1 shows the typical pattern of* H. pylori* antibody titer in subjects who do not show significant progression of damage to the gastric mucosa despite infection with* H. pylori*. This subset of patients is classified as Group B in the ABC method. In most of these cases, antibody titer does not show significant change. However, an increase in* H. pylori *antibody titer is observed in a subset of these cases, specifically those with progressive mucosal inflammation, who have an increased risk for diffuse-type cancer. Thus, the subjects in Group B are subdivided into the following two subgroups: subjects with a low antibody titer (at low risk for future gastric cancer) and subjects with a high antibody titer due to advanced mucosal inflammation (at high risk for future gastric cancer) (Figure 1).

Figure 2 shows the typical pattern of* H. pylori* antibody titer in subjects who show significant progressive atrophy of the gastric mucosa. This subset of patients is classified as Group C in the ABC method. In some cases, the antibody titer will not change significantly (unlike the situation in Group B); however, in other cases, the* H. pylori* antibody titer will decrease gradually due to shrinkage of the effective area of gastric mucosa by* H. pylori* infection, although the degree of atrophy will differ depending on the pathogenicity of specific* H. pylori* strains and the immunological response of each human host. Subjects with a lower* H. pylori* IgG titer have a higher risk for gastric cancer than high-positive titer subjects in Group C, as reported by Tatemichi et al. [5]. We have confirmed this pattern in our own work investigating the association between gastric acidity and* H. pylori* antibody titer [54].

Figure 3 shows the typical pattern of the* H. pylori* antibody titer in subjects who show significant “advanced” progression of atrophy of gastric mucosa, with the* H. pylori* antibody titer becoming negative following the spontaneous disappearance of* H. pylori* from the gastric mucosa. This subset of patients is classified as Group D in the ABC method. About 99% of* H. pylori*-infected patients are classified into Groups A, B, and C; the remaining approximately 1% of* H. pylori*-infected cases are defined as belonging to Group D. Specifically, separate reports by Miki and Yoshida et al. reported the incidence of Group D as 0.66% (33 of 5209 subjects) and 0.71% (33 of 4655 subjects), respectively [1, 8]. A limited number of* H. pylori*-infected patients show extremely deep progression of atrophy with negative conversion of* H. pylori* antibody titer; most of these cases are hypothesized to originate as low antibody titer Group C subjects. Members of this class show high risk for gastric carcinogenesis, especially differentiated adenocarcinoma.

Figure 4 shows the typical pattern of the* H. pylori* antibody titer in subjects from whom* H. pylori* has been eradicated, irrespective of whether elimination resulted from treatment with conventional triple therapy or by “unexpected” eradication. This subset of patients is sometimes misclassified as Group A in the ABC method; however, the risk for the development of gastric cancer is comparable to that in Group B. These subjects are not true-negatives for* H. pylori* and thus should be distinguished from Group A. We have demonstrated [54] that a high-negative* H. pylori* antibody titer is a significant predictor of gastric neoplasia in Group A cases. We advocate lowering the cut-off value for the* H. pylori* antibody titer, a step that would permit detection of such “eradicated” cases.

## 6. Modified Schematic Diagram of the ABC Method Considering the Significance of the* H. pylori *Antibody Titer

**Figure 5:**
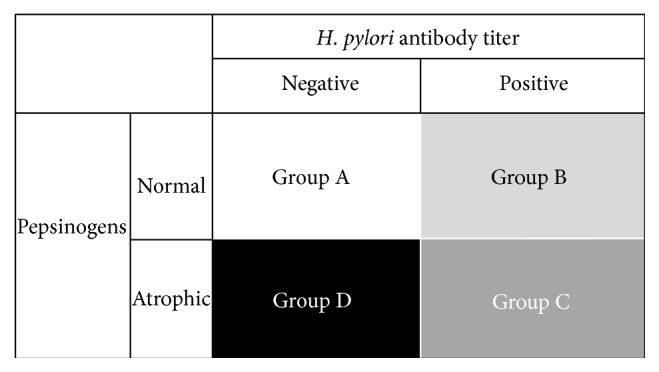
ABC method classification by* H. pylori* serology and pepsinogen status. Subjects are divided into four groups according to the serology of* H. pylori *antibody and pepsinogen (PG) status: Group A [*H. pylori* (−) PG (−)], Group B [*H. pylori* (+) PG (−)], Group C [*H. pylori* (+) PG (+)], and Group D [*H. pylori* (−) PG (+)]. The incidence of gastric cancer increases in a stepwise and significant manner from A to D. The color gradation shows the risk for gastric cancer.

Figure 5 shows the generally accepted classification of the ABC method, in which the cases are divided into the following four groups: Group A [*H. pylori* (−) PG (−)], Group B [*H. pylori* (+) PG (−)], Group C [*H. pylori* (+) PG (+)], and Group D [*H. pylori* (−) PG (+)]. The incidence of gastric cancer increases in a stepwise and significant manner from A to D, as the color gradation shows the increasing risk for gastric cancer.

**Figure 6:**
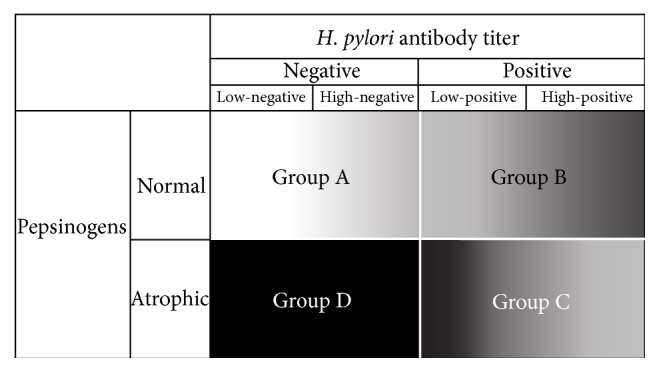
Modified schematic diagram of the ABC method considering the antibody titer as a continuous variable. Groups A, B, and C can each be divided into two subgroups based on antibody titer. The color gradation shows schematically the risk for gastric cancer. In Group A, the incidence of gastric cancer in the high-negative antibody subset is higher than that in the low-negative antibody titer subset. In Group B, the incidence of gastric cancer (typically diffuse-type cancer) in the high-positive titer antibody subset is higher than that in the low-positive titer antibody subset. In Group C, the HR of gastric cancer (typically intestinal-type cancer) is higher in low-positive titer subjects than in high-positive titer subjects.

Figure 6 shows a modified schematic diagram of the ABC method incorporating the antibody titer as a continuous variable, as proposed in this review. The color gradation shows the risk for gastric cancer. The horizontal axis showing* H. pylori* serology is transformed from a categorical variable (i.e., positive or negative) to a continuous variable of antibody titer. For Group A, we demonstrated that the incidence of intestinal-type gastric neoplasia in the high-negative antibody subset was higher than in the low-negative antibody titer subset [54]. For Group B, the incidence of diffuse-type gastric cancer in the high-titer antibody subset was elevated compared to the cases that were* H. pylori*-positive but possessed low antibody titer [7]. For Group C, the HR of intestinal-type gastric cancer was higher in low-titer subjects than in high-titer subjects.

## 7. Conclusions

Clinicians should consider the meaning of the antibody titer when interpreting a patient's status as defined by the ABC method. The risk for gastric cancer in subjects classified into each group is not the same, but clinicians should understand the fact that some high-risk populations detected by antibody titer stratification are included. We recommend dividing the cases by antibody titer into four subgroups, not two (negative and positive) subgroups: low-negative, high-negative, low-positive, and high-positive. One example of the range of each subgroup is <3 U/mL, 3–10 U/mL, 10–50 U/mL, and >50 U/mL, respectively. We especially advocate attention to the antibody titer itself in the following three clinical conditions.

The first condition occurs when a high-negative antibody titer is observed in PG-negative cases (Group A). Most of these subjects are posteradicated cases who have an elevated risk for developing gastric cancer. Lowering the cut-off value of the* H. pylori* antibody titer to the lower limit of each EIA kit will be important for the detection of these high-risk eradicated cases.

The second condition occurs when a high-positive antibody titer is observed in PG-negative cases (Group B). These subjects exhibit active mucosal inflammation and have an elevated risk for developing diffuse-type cancer.

The third condition occurs when positive but low-positive antibody titer is observed in subjects with mucosal atrophy (as defined by PG levels) (Group C). These subjects have severe progressive atrophy with a high risk for intestinal-type gastric cancer. The cutoff values of low and high-positive antibody titers should be set at values that put about half of the seropositive subjects into each subgroup.

In these cases, we consider that the interval of endoscopic surveillance should be shorter than the interval recommended by the ABC method: every three years for subjects with a high-negative antibody titer in Group A; every two years for subjects with a high-positive antibody titer in Group B; and every year for subjects with a low-positive antibody titer in Group C. Moreover, eradication of* H. pylori* is also strongly recommended to prevent future development of gastric cancer. Even in subjects who are serologically negative but have a high-negative antibody titer (Group A),* H. pylori* should be evaluated by other methods, and it should be eradicated if the results of the other evaluation methods are positive.

There is a possibility that gastric cancer screening solely by the ABC method is insufficient for the above-mentioned limited number of cases detected only by considering antibody titer. We believe that focusing on the serum* H. pylori* antibody titer as a quantitative (rather than qualitative/categorical) parameter will enhance the power of the ABC method as a screen for future risk for gastric cancer.
